# Estimating intracranial volume using intracranial area in healthy children and those with childhood status epilepticus

**DOI:** 10.1002/brb3.271

**Published:** 2014-08-28

**Authors:** Rory J Piper, Michael M Yoong, Suresh Pujar, Richard F Chin

**Affiliations:** 1 College of Medicine and Veterinary Medicine, University of EdinburghEdinburgh, U.K.; 2 Muir Maxwell Epilepsy Centre, University of EdinburghEdinburgh, U.K.; 3 Department of Paediatric Neurosciences, Royal Hospital for Sick ChildrenEdinburgh, U.K.; 4 Institute of Child Health, University College LondonLondon, U.K.

**Keywords:** Epilepsy, intracranial area, intracranial volume, pediatric, volumetric

## Abstract

**Background:**

Correcting volumetric measurements of brain structures for intracranial volume (ICV) is important in comparing volumes across subjects with different ICV. The aim of this study was to investigate whether intracranial area (ICA) reliably predicts actual ICV in a healthy pediatric cohort and in children with convulsive status epilepticus (CSE).

**Methods:**

T1-weighted volumetric MRI was performed on 20 healthy children (control group), 10 with CSE with structurally normal MRI (CSE/MR-), and 12 with CSE with structurally abnormal MRI (CSE/MR+). ICA, using a mid-sagittal slice, and the actual ICV were measured.

**Results:**

A high Spearman correlation was found between the ICA and ICV measurements in the control (*r* = 0.96; *P *< 0.0001), CSE/MR− (*r* = 0.93; *P* = 0.0003), and CSE/MR+ (*r* = 0.94; *P *< 0.0001) groups. On comparison of predicted and actual ICV, there was no significant difference in the CSE/MR− group (*P* = 0.77). However, the comparison between predicted and actual ICV was significantly different in the CSE/MR+ (*P* = 0.001) group. Our Bland–Altman plot showed that the ICA method consistently overestimated ICV in children in the CSE/MR+ group, especially in those with small ICV or widespread structural abnormalities.

**Conclusions:**

After further validation, ICA measurement may be a reliable alternative to measuring actual ICV when correcting volume measurements for ICV, even in children with localized MRI abnormalities. Caution should be applied when the method is used in children with small ICV and those with multilobar brain pathology.

## Introduction

Considering the variation in intracranial volume (ICV) across brain development (Courchesne et al. 2000), volumes-of-interest calculated in studies of pediatric subjects require correction for ICV. This is of particular importance when comparing volumetric data obtained from pediatric subjects of different age or when conducting follow-up, longitudinal studies (Whitwell et al. 2001). For example, correction for ICV was important in our recent study on magnetic resonance imaging (MRI) changes in children following epileptic seizure activity lasting at least 30 min, convulsive status epilepticus (CSE) (Yoong et al. 2012).

The measurement of actual ICV requires either laborious manual segmentation or advanced automated image processing, which may be expensive, requires training in quantitative neuroimaging and may not be widely available for use in clinical centers. Furthermore, in clinical practice, lower resolution MRI scanners (1.5 Tesla) are often used and increased slice thickness may complicate either automated or manual full-brain ICV measurement (Nandigam et al. 2007). Thus, an efficient, simple, and accessible method of assessing ICV is needed. Such a method may allow clinicians to easily and quantitatively assess the longitudinal brain development of pediatric patients using widely available clinical imaging platforms, such as PACS (Kodak Health Information Systems, Dallas, TX; http://www.carestream.com/).

Ferguson et al. designed and validated a simple MRI segmentation method that employs a measure of intracranial area (ICA) as an indicator of ICV in an adult cohort (Ferguson et al. 2005). The method requires only a single midsagittal slice in which the cranial cavity is delineated. The investigators found that ICA was highly correlated with ICV (Pearson *r* = 0.88, *P *< 0.0001, *n* = 40) and consistent across observers. Furthermore, the method requires considerably less processing time than full brain ICV measurement. This method, using only one slice, also allows the prediction of ICV in MRI investigations without complete brain volume coverage.

As argued by Whalley and Wardlaw (2001), the longest dimension or cross-sectional area of an object is the most reliable area that can be used to predict the actual volume of an object. We suggest that this hypothesis is true for the brain also, whereby the ICV can crudely be considered as a sphere. The largest cross-sectional area in the midline (the midsagittal slice) of the sphere theoretically has the same radius as the sphere (the actual ICV) and will, therefore, have an area that correlates with the total volume. While the brain with its coverings is not strictly spherical, the correlation between predicted and actual ICV found by Ferguson et al. (2005) was remarkably accurate.

However, to our knowledge, this method has only been validated in healthy adult volunteers, and a single study of intracranial hemorrhage in adults (Nandigam et al. 2007). Within the published literature, studies of various other conditions that use volumetric analysis have used a measurement of midsagittal area to control for ICV, including temporal lobe epilepsy (Free et al. 1995), small vessel disease (Rost et al. 2010), and Alzheimer's disease (Schofield et al. 1995). To date, this technique has not been validated in pediatric subjects.

Therefore, the aim of this study was to investigate whether ICA correlates with ICV using three groups of children recruited from our previous study investigating the MR consequences of CSE (Yoong et al. 2012). The groups investigated and the reason for their selection in this study are as follows:

1. Healthy and normally developing children to validate the method in healthy children (control group);
2. Children with CSE but with qualitatively diagnosed, structurally normal brain MRI to examine the method in “normal” appearing brain MRIs (CSE/MR− group);
3. Children with CSE with qualitatively diagnosed, structurally abnormal brain MRI to investigate the limits of the technique in the presence of gross pathology (CSE/MR+ group).

## Material and Methods

Twenty randomly selected healthy and normally developing children (11 male and nine female; age range, 0.5–11.6 years; median age, 2.9 years at the time of image acquisition) were recruited as controls. These children had no known history of seizures or neurological illness. They were recruited through internal electronic and printed advertisements in the recruiting centers, by patient-suggested peers, and/or were patient siblings. Children with CSE were recruited as part of a previously published study (Yoong et al. 2012). For the CSE/MR- group, ten children (three male and seven female; age range, 0.9–3.9 years; median age = 1.8 years at the time of image acquisition) with a specific form of CSE associated with fever, prolonged febrile seizure (PFS), and with no structural lesion on their MRI were selected for this cohort. Finally, the CSE/MR+ group included 12 children (10 male and two female; age range, 0.2–7.4 years; median age = 2.1 years at the time of image acquisition) who had previously suffered an episode of symptomatic status epilepticus and had major structural abnormalities on their MRI scan (Table 1). Four children from the CSE/MR+ group had localized pathology (confined to one lobe) and the remaining eight children had widespread pathology (involving more than one lobe). Two experienced pediatric neuroradiologists qualitatively reviewed the MRI data for all of the children. The demographics for all the groups are summarized in Table 2.

**Table 1:** Demographic, quantitative imaging and clinical data for subjects with convulsive status epilepticus and abnormalities on magnetic resonance imaging (CSE/MR+).

Patient ID	Age at scan (years)	ICA (mm^2^)	Predicted ICV (mm^3^)	Actual ICV (mm^3^)	Predicted—actual ICV (mm^3^)	Prediction error (predicted —actual/actual ^*^ 100) (%)	Pathology
1	1.3	11105	956235	821555	134680	16.39	Neonatal hypoxic-ischemic encephalopathy with persistent bilateral white matter abnormality
2	1.6	15433	1419331	1423687	4356	0.31	Ventriculo-peritoneal shunt in situ following previous choroid plexus resection
3	0.6	9576	792632	521299	271333	52.05	Extensive damage following neonatal hypoxic-ischemic encephalopathy
4	2.0	11597	1008879	728761	280118	38.44	Extensive damage following neonatal hypoxic-ischemic encephalopathy
5	2.1	12583	1114381	939726	174655	18.59	Polymicrogyria and other extensive congenital brain malformations
6	3.4	13300	1191100	1172103	18997	1.62	Preterm white matter injury and germinal matrix hemorrhage
7	3.4	14058	1272206	1144078	128128	11.20	Left parieto-occipital infarction following neonatal meningitis
8	2.3	13423	1204261	1162203	42058	3.62	Orbitofrontal cortical dysplasia
9	7.4	15378	1413446	1366937	46509	3.40	Right mesial temporal sclerosis
10	5.3	15731	1451217	1310962	140255	10.70	Ventriculo-peritoneal shunt in situ following neonatal hemorrhagic hydrocephalus
11	0.2	9006	731642	635655	95987	15.10	Tuberous sclerosis
12	0.3	6755	490785	469181	21604	4.60	Postmeningitis

ID, identification; ICA, intracranial area; ICV, intracranial volume.

**Table 2:** Subject demographics, intracranial area (ICA), and intracranial volume (ICV) ranges and median values, correlation value (Spearman's test) of all three groups (control; convulsive status epilepticus with no abnormalities on magnetic resonance imaging (CSE/MR−); convulsive status epilepticus with abnormalities on magnetic resonance imaging (CSE/MR+).

Group	*N*	Gender	Age range (median) in years	ICA range (median) in mm^2^	ICV range (median) in mm^3^	Correlation between ICA and ICV
Control	20	11 M, 9 F	0.5–11.6 (2.9)	10166–17314 (14430.5)	825163–1624496 (1318746)	*r* = 0.96; *P* < 0.0001
CSE/MR−	10	3 M, 7 F	0.9–3.9 (1.8)	11540–14890 (12804)	979111–1398082 (1192463)	*r* = 0.93; *P* = 0.0003
CSE/MR+	12	10 M, 2 F	0.2–7.4 (2.1)	6755–15731 (12942)	469181–1423687 (1041902)	*r* = 0.94; *P* < 0.0001

All MRI investigations were performed on the same 1.5-T scanner (Siemens AG, Muenchen, Germany) using a protocol including a T1-weighted Fast Low Angle Shot (3-D FLASH) sequence (repetition time = 4.94 ms, echo-time = 11 ms, acquisition matrix 256 × 224, in-plane resolution 1.0 × 1.0 mm, slice thickness 1 mm).

**Figure 1:**
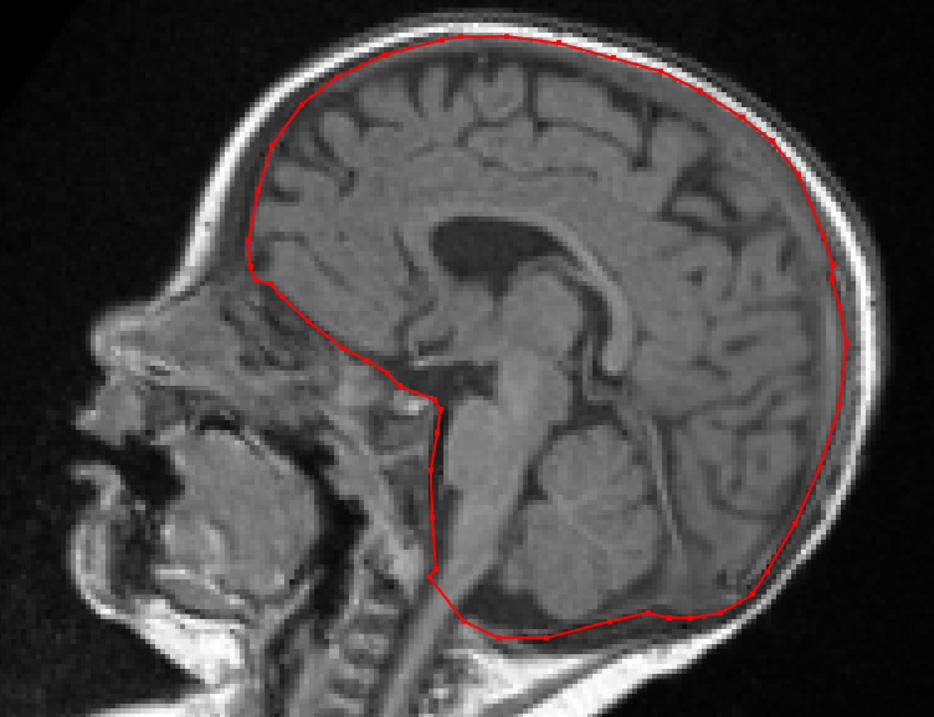
Example of intracranial area (ICA) segmentation in a child from the control group.

Intracranial area was measured using the manual segmentation method as employed by Ferguson et al. (2005). In a midline sagittal view, a region of interest was traced around the inner table of the cranial vault, along the frontal fossa floor, across the pituitary fossa at the dorsum sella, down the posterior surface of the clivus and horizontally across the foramen magnum. The midline slice was selected using two criteria; one, that all the aforementioned anatomical features were present and two, that it was selected from between the cerebral hemispheres. Manual segmentation of the ICA was conducted on ITK-SNAP software (Yushkevich et al. 2006) (http://www.itksnap.org/). Where the sagittal sections were in a significant oblique plane, such that the anatomical features needed for segmentation, as described, were not found in the same slice, scans were resliced into a true sagittal plane using MRIcro (http://www.mccauslandcenter.sc.edu/mricro/). An example of the ICA segmentation is demonstrated in Fig.1.

Actual ICV was calculated by semiautomated segmentation, using the *brain extraction tool* in FSL (Smith 2002) with an intensity threshold of 0.3. In each image of the volume, the intracranial brain tissue was segmented from the surrounding skull and overlying tissue. Each image was manually inspected adjusted to minimize segmentation error.

Measurement of ICA and ICV were conducted independently by two researchers trained in quantitative neuroimaging (RJP measured ICA, and MY measured ICV), each of who were blinded to the clinical details and the other investigator's measurements. Spearman's test was used to analyze the correlation between ICA and ICV in all groups. Linear regression was used to define the relationship between ICA and ICV for each of the patients using data from the control group. The linear relationship thus derived was used to generate predicted values of ICV from ICA measurements obtained in the CSE/MR- and CSE/MR+ groups. Using the Wilcoxon matched pairs test, predicted and actual ICV values were compared in each group and Bland–Altman plotting was used to search for systematic errors. The distance between predicted and actual ICV values was compared within the CSE-MR+ cohort in children with localized pathology and those with widespread pathology. In addition, MY measured ICA in 10 of the subjects in the control group for the interrater analysis. Intrarater and interrater comparisons of ICAs were analyzed using Spearman's test. Distribution of ICV across the groups was assessed using the Kruskal–Wallis test and post hoc Dunn's multiple comparison test.

As a final analysis, we compared our regression equation to that derived by Ferguson et al. (2005) (ICA*99.9 + 5479.8). In the control group, we used each equation to predict ICV and compared the results to actual ICV.

Statistical analyses were carried out using GraphPadPrism v5.0 (GraphPad Software Inc, CA, USA; http://www.graphpad.com) for MacOSX. Statistical significance was set at *P* < 0.05 and data are reported to two significant statistical figures.

Informed consent was taken from all parents of children involved in the original study that was approved by the Great Ormond Street Hospital (GOSH) research ethics committee.

## Results

Each individual ICA region took <5 min to segment and quantify using simple manual segmentation. Actual ICV measurement from full brain manual segmentation, as employed in our previous investigations, takes on average 30 min for each subject. Semiautomated ICV measurements, as employed to measure ICV in this study, took a similar time (around 5 min) to measuring ICA. However, checking for error after semiautomated segmentation was required (1-2 min) and when semiautomated segmentation did not work further manual adjustments were required (15 min). Manual adjustment was required after semiautomated ICV measurement in 2/20 control, 0/10 CSE/MR- and 5/12 CSE/MR+ children.

**Figure 2:**
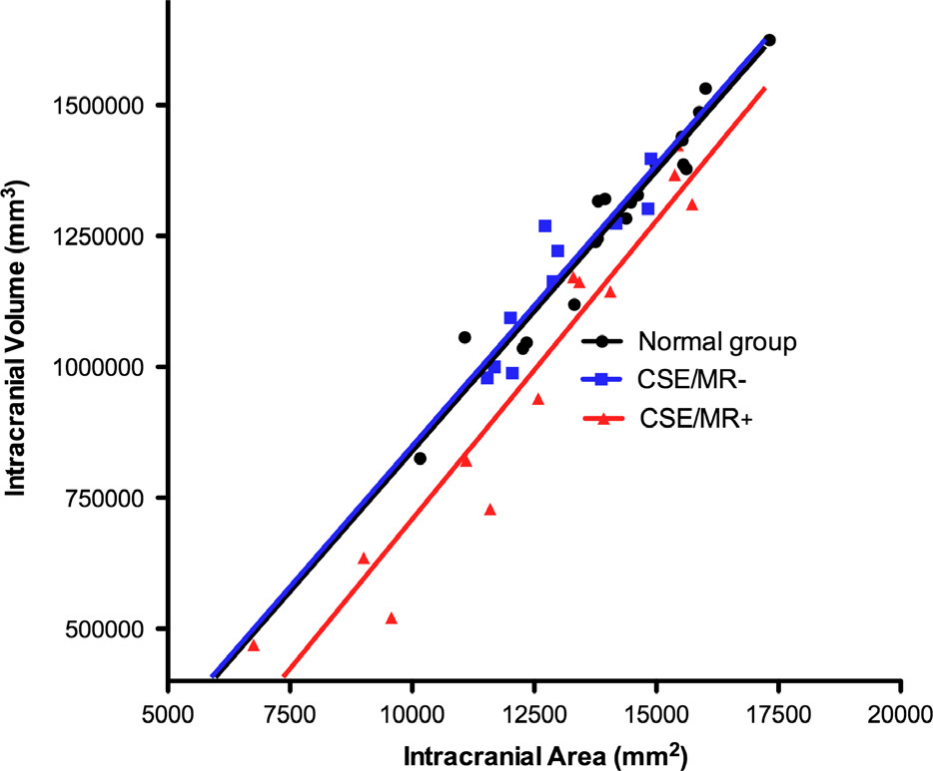
Intracranial area (ICA) vs. intracranial volume (ICV) in controls (Spearman's test: *r* = 0.96; *P* < 0.0001; *n* = 20), children with convulsive status epilepticus with no abnormalities on magnetic resonance imaging (CSE/MR−) (Spearman's test: *r* = 0.93; *P *< 0.0003; *n* = 10) and children with convulsive status epilepticus with abnormalities on magnetic resonance imaging (CSE/MR+) (Spearman's test: *r* = 0.94; *P* < 0.0001; *n* = 12) groups. Linear regression in the control group: ICV=107*ICA–232000.

**Figure 3:**
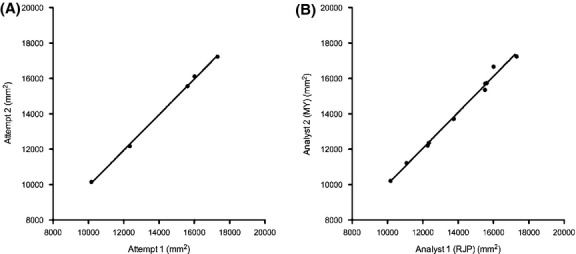
(A) Intrarater analysis for intracranial area (ICA) measurement in the control group (Spearman's test: *r* = 1.0; *P* = 0.017). (B) Interrater analysis for ICA measurement in the control group (Spearman's test: *r* = 1.0; *P* < 0.0001).

Intracranial area measurements in the control group ranged from 10166–17314 mm^2^ (median = 14430.5 mm^2^) and the ICV measurements from 825163–1624496 mm^3^ (median=1318746 mm^3^). A high Spearman correlation was found between the ICA and ICV measurements (*r* = 0.96; *P* < 0.0001, *n* = 20) (Fig.2). The linear regression equation was predicted: ICV = 107*ICA–232000. Repetition of the measurement of ICA in five randomly selected subjects by one analyst (RJP) showed an intrarater correlation of *r* = 1.0 (*P* < 0.017) with the initial measurements (Fig.3A). Measurements of ICA in ten randomly selected subjects by a second analyst (MY) blinded to the results from the initial analyst showed an interrater correlation of *r* = 1.0 (*P* < 0.0001) (Fig.3B).

**Figure 4:**
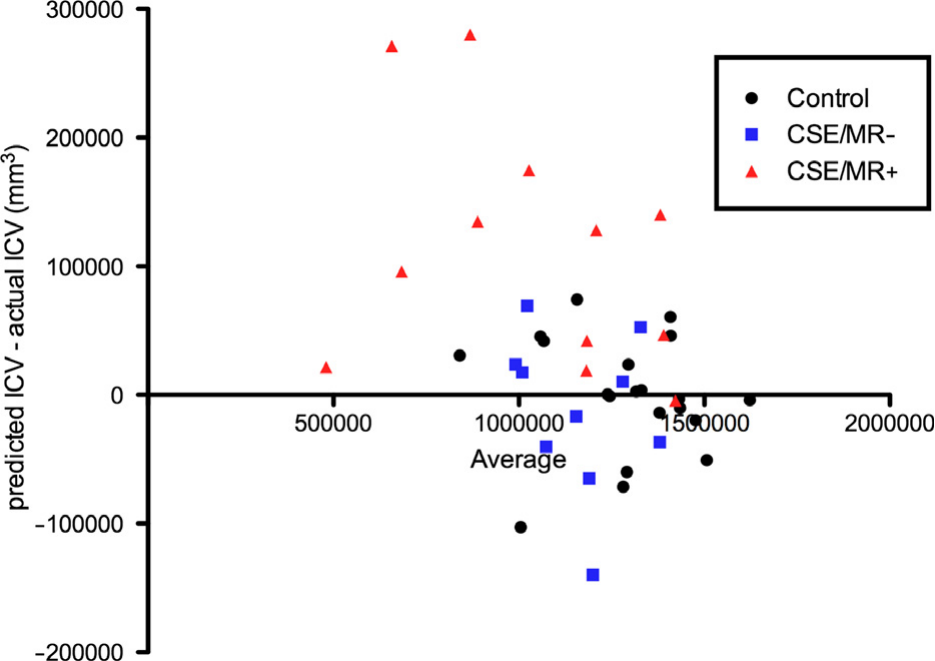
Bland–Altman graph of predicted ICV—actual ICV for the control, CSE-MR− and CSE-MR+ group.

In the CSE/MR- group, ICA measurements ranged from 11540–14890 mm^2^ (median = 12804 mm^2^) and the ICV measurements from 979111–1398082 mm^3^ (median = 1192463 mm^3^). ICA and ICV measurements were highly correlated (*r* = 0.93; *P* = 0.0003, *n* = 10) (Fig.2). Predicted and actual ICV in this group were not statistically different (*P* = 0.77), as shown in the Bland–Altman plot in Fig.4.

Intracranial area measurements in the CSE/MR+ group with structurally abnormal MRIs scans ranged from 6755–15731 mm^2^ (median = 12942 mm^2^) and the ICV measurements from 469181–1423687 mm^3^ (median = 1041902 mm^3^). On group analysis, there was a high Spearman correlation between the ICA and ICV measurements (*r* = 0.94; *P* < 0.0001, *n* = 12) (Fig.2). At the group level, predicted and actual ICV were statistically different (*P* = 0.001) and a greater difference between predicted and actual ICV was seen in subjects with smaller actual ICV. Our Bland–Altman plot reveals a consistent overestimation of ICV in the CSE/MR+ group, particularly in children with small ICV (Fig.4). The distance between the predicted and actual ICV was greater for those CSE/MR+ children with widespread structural abnormalities than CSE/MR+ children with localized lesions, but this was not statistically significant (*P *= 0.13).

Using the Kruskal–Wallis test and post hoc Dunn's multiple comparison test, distribution of ICV across the groups was significantly different since the ICV in the CSE/MR+ group was significantly smaller.

When Ferguson's equation was applied in the control group it overestimated each ICV by >500000 mm^3^ (range, 55960–217095 mm^3^), and was less accurate than our regression in 18/20 cases.

The values for ICA, ICV, and the correlation analyses are summarized in Table 2.

## Discussion

Our findings suggest that using ICA as a predictor of ICV is a valid method not just in healthy children (controls), but even in children who have had prolonged epileptic seizures, irrespective of whether their MRI were qualitatively normal or abnormal with localized pathology. The method is less accurate in patients with smaller ICV and this is concurrent with the findings by Whalley and Wardlaw (2001) who found a poor correlation between cross-sectional area and actual volume in smaller objects. This is likely due to the fact that the area will be proportional to *r*^2^, while the volume to *r*^3^. While this can be approximated by a linear relationship for small variations in r, it may not be surprising that it is less accurate at the extremes of measurement. Nevertheless, we have demonstrated that this remains a reasonable approximation for the majority of physiological values of ICV/ICA.

When Ferguson's equation (Ferguson et al. 2005) was applied in the control group it overestimated each ICV by >500000 mm^3^. Ferguson's equation was more accurate than our method in only two cases. Thus, it appears that our regression equation for ICA and ICV is more accurate in children than Ferguson's (validated in 65–70 year old men). However, further validation of this regression equation is required in children of different ages, populations, MR scanners, and protocols. The difference in the results generated by the equations may be explained by the fact that we apply a linear approximation to a nonlinear relationship, and therefore the most accurate approximation of ICV will differ depending on the brain size of the population.

Data from the current study indicate that caution should be taken when applying the method in subjects who have pathology in more than one brain lobe in whom the method may be less accurate. The two largest prediction errors (predicted–actual/actual * 100) of the ICA method, 38.44% and 52.05%, were found in two patients both with extensive damage following neonatal hypoxic-ischemic encephalopathy.

Our study shows that estimating ICV using ICA measurement on a single sagittal slice through manual segmentation is faster than full-brain ICV semiautomated segmentation. When it worked, advanced automated methods of ICV segmentation was completed in a similar time to the ICA method. However, automated methods often required manual editing and validation of their MRIs because in younger subjects tissue contrast differs to that of adults (Evans 2006). Therefore, the ICA method put forward here may be an overall faster alternative to full-brain ICV segmentation. In research, this would allow the efficient estimation of ICV when large numbers of subjects are included. A further advantage is that, as it only requires a midline sagittal slice, this method can be performed on older MRI sequences that do not feature full brain coverage.

Furthermore, as detailed, the relatively simple manual segmentation and quantification were performed using freely available software, avoiding computationally expensive and complex fully automated segmentation. Thus, this technique can be performed with manual region-of-interest analyses on widely used clinical imaging platforms, such as PACS. This method could be used clinically for assessing longitudinal brain development and to quantify structural lesions controlled for variation in ICV. This method was successfully performed using PACS in a previous study by R. J. Piper, H. Blackwood, S. Kerrigan, J. L. Scotland, T. Carpenter and I. R. Whittle. (unpubl. data). It could be argued that the software (such as *Brain Extraction Tool*, on FSL) (Smith 2002) required to run automated methods of ICV calculation could be installed on clinical systems. However, the ICA method described holds the advantages that it can run on existing UK-wide National Health Service clinical technology and it does not require extensive training. MR images with exceptional artifact or disruption may preclude full brain ICV measurement, however this problem may be avoided witht the ICA method since it requires only one slice.

This quantitative method may offer clinicians the capability to assess the longitudinal brain development of children. Measuring ICA may also offer an alternative to correcting for ICV in the quantitative assessment of regional brain volumes, such as the temporal lobe. However, further validation is required.

Other limitations of the method may be that, compared with automated methods, manual measurement of ICA may allow for bias. Also, even minimal predictive error between ICA and ICV may hold significant differences on correcting values for structures with small volume such as the hippocampi. The ICA method may also capture CSF in the area measured and thus may overestimate the intracranial volume, especially in cases of acquired brain atrophy.

Although we have adopted the Whalley and Wardlaw (2001) concept that the longest dimension or cross-sectional area of an object is the most reliable indication of the actual volume, the brain is not a sphere. Therefore, predicting ICV on the basis of the longest cross-section of the brain (midsagittal slice) will likely overestimate ICV. This was evidently a problem in children in the CSE-MR+ group, and is shown in the Bland–Altman plot (Fig.4). Our method may have also consistently overestimated ICV in the CSE-MR+ group for the following reasons: abnormal head shape, damage to/reduction in size of nonmidline structure which would not have been captured on a midsagittal ICA, and nonuniform growth.

## Conclusions

To our knowledge, this is the first study to demonstrate that ICA can be used to predict ICV in MRIs of the developing human brain. Our findings suggest that the use of this method is valid not just in healthy children (controls), but even in children who had prolonged epileptic seizures irrespective of whether their MRI were qualitatively normal or qualitatively abnormal with localized pathology. Caution should be applied when the method is used in children with small ICV and those with multilobar brain pathology. We have demonstrated the utility of a highly reproducible, efficient and accurate technique that allows for the estimation of ICV for individual subjects based on that individual's own ICA and does not rely on any other group analyses.
